# Novel Double-Hit Model of Radiation and Hyperoxia-Induced Oxidative Cell Damage Relevant to Space Travel

**DOI:** 10.3390/ijms17060953

**Published:** 2016-06-16

**Authors:** Ralph A. Pietrofesa, Anastasia Velalopoulou, Stacey L. Lehman, Evguenia Arguiri, Pantelis Solomides, Cameron J. Koch, Om P. Mishra, Constantinos Koumenis, Thomas J. Goodwin, Melpo Christofidou-Solomidou

**Affiliations:** 1 Division of Pulmonary, Allergy, and Critical Care Medicine and the Department of Medicine, University of Pennsylvania Perelman School of Medicine, 3450 Hamilton Walk, Edward J. Stemmler Hall 2nd Floor, Office Suite 227, Philadelphia, PA 19104, USA; ralphp@mail.med.upenn.edu (R.A.P.); avela@mail.med.upenn.edu (A.V.); evguenia@mail.med.upenn.edu (E.A.); pantelis.solomides@temple.edu (P.S.); mishra.o@gmail.com (O.P.M.); 2 Department of Radiation Oncology, University of Pennsylvania Perelman School of Medicine, Philadelphia, PA 19104, USA; staceylehman87@gmail.com (S.L.L.); kochc@mail.med.upenn.edu (C.J.K.); costas.Koumenis@uphs.upenn.edu (C.K.); 3 The National Aeronautics and Space Administration (NASA) Johnson Space Center, Houston, TX 77058, USA; tgoodwin3@comcast.net

**Keywords:** cell cycle, DNA damage, extravehicular activity, hyperoxia, ionizing radiation, lung cell injury, oxidative stress, reactive oxygen species, and space exploration

## Abstract

Spaceflight occasionally requires multiple extravehicular activities (EVA) that potentially subject astronauts to repeated changes in ambient oxygen superimposed on those of space radiation exposure. We thus developed a novel *in vitro* model system to test lung cell damage following repeated exposure to radiation and hyperoxia. Non-tumorigenic murine alveolar type II epithelial cells (C10) were exposed to >95% O_2_ for 8 h only (O_2_), 0.25 Gy ionizing γ-radiation (IR) only, or a double-hit combination of both challenges (O_2_ + IR) followed by 16 h of normoxia (ambient air containing 21% O_2_ and 5% CO_2_) (1 cycle = 24 h, 2 cycles = 48 h). Cell survival, DNA damage, apoptosis, and indicators of oxidative stress were evaluated after 1 and 2 cycles of exposure. We observed a significant (*p* < 0.05) decrease in cell survival across all challenge conditions along with an increase in DNA damage, determined by Comet analysis and H2AX phosphorylation, and apoptosis, determined by Annexin-V staining, relative to cells unexposed to hyperoxia or radiation. DNA damage (GADD45α and cleaved-PARP), apoptotic (cleaved caspase-3 and BAX), and antioxidant (HO-1 and Nqo1) proteins were increased following radiation and hyperoxia exposure after 1 and 2 cycles of exposure. Importantly, exposure to combination challenge O_2_ + IR exacerbated cell death and DNA damage compared to individual exposures O_2_ or IR alone. Additionally levels of cell cycle proteins phospho-p53 and p21 were significantly increased, while levels of CDK1 and Cyclin B1 were decreased at both time points for all exposure groups. Similarly, proteins involved in cell cycle arrest was more profoundly changed with the combination challenges as compared to each stressor alone. These results correlate with a significant 4- to 6-fold increase in the ratio of cells in G2/G1 after 2 cycles of exposure to hyperoxic conditions. We have characterized a novel *in vitro* model of double-hit, low-level radiation and hyperoxia exposure that leads to oxidative lung cell injury, DNA damage, apoptosis, and cell cycle arrest.

## 1. Introduction

Extravehicular activities (EVA) are required by astronauts during space travel and may pose potential health risks [1,2,3,4]. Astronauts are exposed to the outer space environment while performing EVAs, including routine maintenance during space flights [5]. During EVAs that typically last for 5–8 h, crewmembers use pressurized spacesuits to create a controlled internal environment [5]. While acute oxygen toxicity is carefully avoided by extensive training, the long term influence of multiple EVAs and ensuing cyclic exposures to hyperoxic and hypoxic conditions amidst hypobaric pressures [6] during pre-breathe training protocols, and thus human exploration missions, on lung function is not known. Considering that there will be an increased requirement for crew members to perform frequent EVAs as deep space exploration becomes a reality, conditions that could pose a risk to the safety and health of the crew must be identified and prevented by designing modified procedure protocols.

Lung complications occurring from prolonged space travel are not well characterized [7]. In addition to space radiation exposure, health risks are compounded by exposure to 100% O_2_. Hyperbaric and hyperoxic conditions have been extensively studied in relation to decompression illness (DCI) in divers [8]. However, exposure to a unique spectrum of space radiation including galactic cosmic radiation (GCR) and solar particle events (SPE) [9,10] adds an additional environmental risk that may affect lung tissue primed by repeat exposures to hyperoxia during preparation for and during exploration EVAs at hypobaric pressures. With the use of a mouse model to identify potential risks to lung tissues when exposed to conditions associated with space travel, we have characterized significant air space enlargement, lung inflammation, and cellular injury [11].

To address the knowledge gap regarding lung complications that result from the combined effects of repeat hyperoxia and radiation exposures, we established an *in vitro* model system to test these effects at the cellular level. We have recently developed a novel *in vivo* mouse model to study individual stressors such as hyperoxia or low levels of radiation exposures as well as the combinatorial effects of both stressors and demonstrated that low level radiation and hyperoxia exposure results in lung inflammation, fibrosis and oxidative tissue damage in mice [12,13]. The present study was designed to develop and characterize an *in vitro* model to investigate the underlying molecular mechanisms of double-hit-induced lung damage using murine pulmonary epithelial cell cultures under controlled atmospheric conditions. Our goal was to use this *in vitro* model to characterize potential pathways of cell damage and death that lead to deleterious changes in lung cells and ultimately impair lung function. Although such an *in vitro* model system lacks the important immune response system of an intact animal, known to contribute to radiation [14] and hyperoxia [15] damage, valuable information can be gained to provide insight to individual cell responses.

We hypothesized that lung epithelial cells exposed to hyperoxia and radiation will experience increased oxidative cell damage resulting from an increased production of reactive oxygen species (ROS) following hyperoxia and radiation exposure. Additionally, we hypothesized that lung epithelial cells exposed to the combined challenge of radiation and hyperoxia will experience increased cellular injury and impairment. In the present study, we evaluated lung epithelial cell viability, DNA damage, apoptosis, and indicators of oxidative stress in an *in vitro* model of radiation and hyperoxia exposure simulating challenges relevant to space travel.

## 2. Results

We have recently developed a novel *in vivo* murine model of repeated double-hit radiation and hyperoxia exposure relevant to space travel to identify potential acute and long term damaging effects in lung [12,13]. To address mechanisms underlying lung cell damage induced by exposure to radiation and hyperoxia, however, we developed an *in vitro* model system that permitted cell exposure to combination radiation and hyperoxia.

### 2.1. Novel Design of Airtight Chambers for in Vitro Exposures to Hyperoxia and Radiation

**Figure 1:**
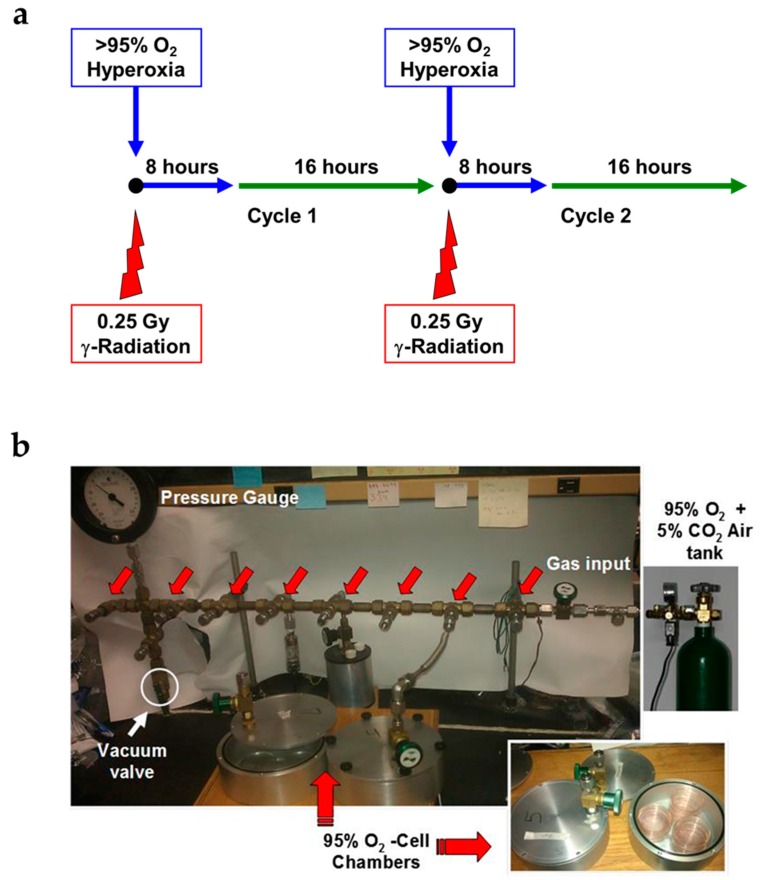
Experimental plan of *in vitro* cell exposure. (**a**) Non-tumorigenic murine alveolar type II epithelial cells (C10) were exposed to 100% O_2_ for 8 h only (O_2_), 0.25 Gy ionizing γ-radiation (IR) only, or a double-hit combination of both challenges (O_2_ + IR) followed by 16 h of normoxia (21% partial pressure of oxygen (pO_2_)) (1 cycle). This was repeated for 2 cycles and cells were harvested at the end of each 24 h cycle: 1 and 2 cycles of exposure corresponding to 24 and 48 h, respectively; (**b**) Images displaying the experimental setup of *in vitro* cell exposure. Pictured are the chambers holding the 6 cm cell culture Petri dishes and the gas manifold allowing for O_2_ exposure.

Select stress conditions to lung tissues such as exposure to high oxygen levels [16] or to radiation [17], result in lung damage; however, there is no cell system that would allow the study of the joint stressor challenge at the cellular level. Repeated, short-duration hyperoxia (8 h), low-level radiation levels (0.25 Gy), or the combination of both challenges in lung epithelial cells was evaluated in a study design (Figure 1a) simulating exposures relevant to challenges encountered during space travel and the performance of multiple extravehicular activities. We used specially-constructed, airtight metal chambers that allowed radiation to penetrate, while keeping cells under controlled oxygen levels (Figure 1b) to simulate airway epithelial cell exposure during multiple, weekly EVAs performed by crewmembers. Cells were exposed to two cycles over the period of 24 h (1 cycle) and 48 h (2 cycles) and evaluated for diverse stress and cell damage biomarkers.

### 2.2. Cell Survival, Apoptosis, and Oxidative DNA Damage in Lung Epithelial Cells Exposed to Hyperoxia, Low-Level Radiation, and Double-Hit Combination Challenge

**Figure 2:**
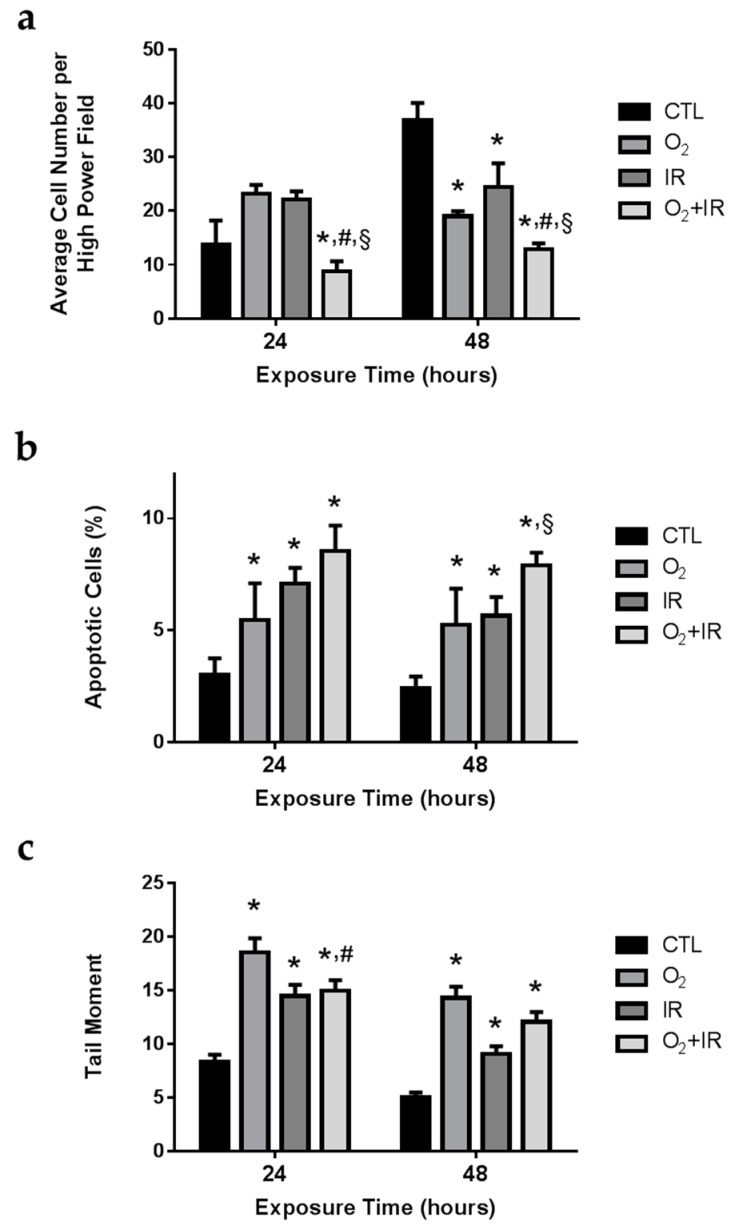
Determination of cell survival, apoptosis, and oxidative DNA damage in lung epithelial cells exposed to hyperoxia, radiation, and double-hit combination challenge. Non-tumorigenic murine alveolar type II epithelial cells (C10) were evaluated after 1 and 2 cycles of hyperoxia and radiation exposure for (**a**) cell survival using light microscopy (200×); (**b**) apoptotic bodies using fluorescent DAPI staining; and (**c**) DNA damage by comet assay analysis. Data are presented as mean ± SEM. * *p* < 0.05 for each exposure condition as it compares with the unexposed control (CTL) in the same respective time-group; ^#^ *p* < 0.05 for significant differences between O_2_-alone compared to O_2_ + IR; ^§^ *p* < 0.05 for significant differences between IR-alone compared to O_2_ + IR.

As anticipated, untreated pulmonary epithelial cells showed an increase in overall cell number after 24 and 48 h of incubation. However, after 2 cycles (48 h) of exposure to challenge conditions, total cell numbers were significantly reduced compared to untreated controls (*p* < 0.05). Overall cell number was significantly (*p* < 0.05) reduced after 1 and 2 cycles of double-hit combination challenge of O_2_ + IR exposure by 36% and 65%, respectively, when compared to controls (Figure 2a).

Murine pulmonary epithelial cells exposed to challenge conditions were analyzed for DNA damage via comet analysis and apoptosis via quantification of apoptotic bodies using fluorescent microscopy. Exposure of lung epithelial cells in the chambers to radiation and hyperoxia significantly (*p* < 0.05) induced oxidative stress, damaged DNA, triggered apoptotic signaling, and ultimately lead to programmed cell death. All challenge conditions (O_2_, IR, O_2_ + IR) significantly induced DNA damage (*p* < 0.05) as measured by comet analysis, which quantifies DNA double strand breaks (Figure 2c). This effect was noted both after 1 cycle (24 h) and 2 cycles (48 h) of exposure. Elevated amounts of irreparable DNA damage led to an increase in apoptosis (*p* < 0.03), measured by the quantification of apoptotic bodies, observed after 1 and 2 cycles of hyperoxia and radiation exposure (Figure 2b). Double-hit (O_2_ + IR) combination challenge significantly (*p* < 0.05) increased the percentage of apoptotic cells from 2% to 8%. We observed exposure-induced reductions in overall cell survival that correlate with increased DNA damage and subsequent apoptosis.

### 2.3. Double-Hit Combination Challenge Switches Cell Death to Apoptosis in Lung Epithelial Cells

**Figure 3:**
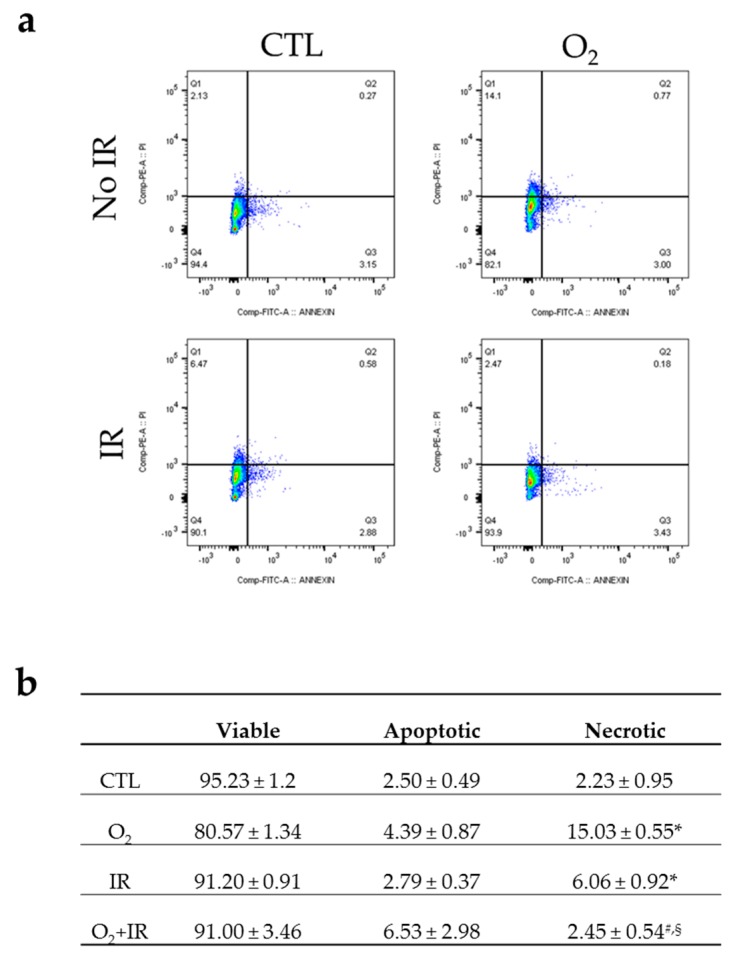
Flow cytometric analysis of apoptosis/necrosis stained with Annexin V-FITC and PI in lung epithelial cells 48 h (2 cycles) after exposure to hyperoxia, radiation, and double-hit combination challenge. Non-tumorigenic murine alveolar type II epithelial cells (C10) were evaluated after 2 cycles of hyperoxia and radiation exposure and evaluated by flow cytometric analysis of apoptosis/necrosis stained with Annexin V-FITC and PI. (**a**) Representative flow cytograms depicting Annexin V-FITC and PI staining showing the distribution of necrotic and apoptotic cells; (**b**) Percentages (%) of apoptotic (positive Annexin V-FITC) and necrotic (negative Annexin V-FITC and positive PI) cells in every cohort, as compared to untreated cells. Data are represented as the mean ± SEM. * *p* < 0.05 for each exposure condition as it compares with the unexposed control (CTL); ^#^ *p* < 0.05 for significant differences between O_2_-alone compared to O_2_ + IR; ^§^ *p* < 0.05 for significant differences between IR-alone compared to O_2_ + IR.

We investigated the cell death induced in non-tumorigenic murine alveolar type II epithelial cells 48 h (2 cycles) after exposure to hyperoxia, low-level irradiation and double-hit combined challenges using Annexin V-FITC and PI staining (Figure 3). Hyperoxia induced a statistically significant (*p* < 0.001) increase in necrotic cell death compared to untreated control (from 2.23% to 15.03%) whereas the percentage of necrotic cells in the irradiated cohort were 6.06% (Figure 3a,b). Interestingly, the double-hit combined exposure to hyperoxia and irradiation switched the profile of cell death from necrosis to apoptosis, as cells exposed to both challenges showed 6.53% of apoptosis when necrosis was maintained at 2.45%, close to baseline levels of the untreated cells (Figure 3b).

### 2.4. Alterations in Cell Cycle Progression in Lung Epithelial Cells Exposed to Repeat Cycles of Hyperoxia, Radiation, and Double-Hit Combination Challenge

**Figure 4:**
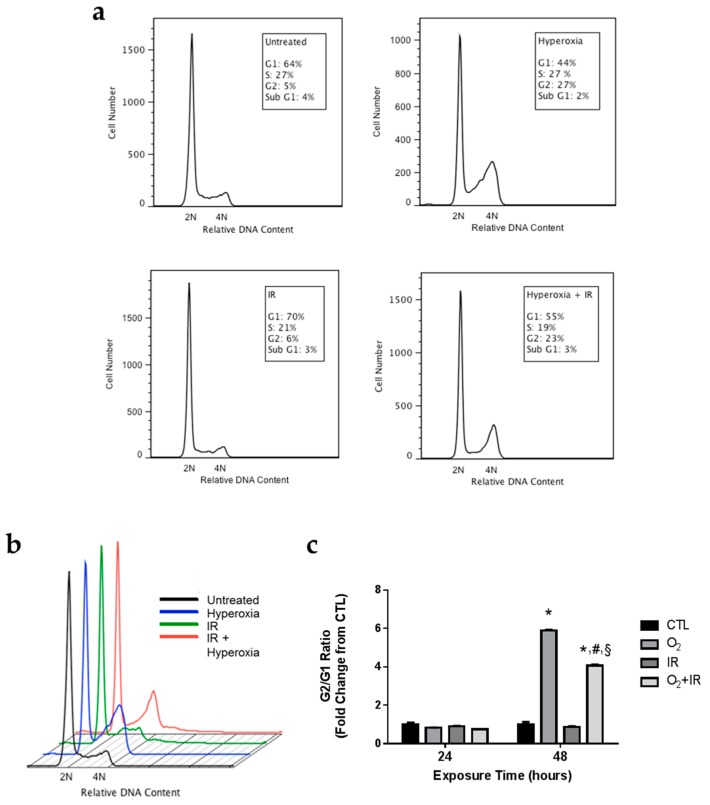
Hyperoxia and Combination Exposure Induces Cell Cycle Arrest. Non-tumorigenic murine alveolar type II epithelial cells (C10) were evaluated after 1 and 2 cycles of hyperoxia and radiation exposure and evaluated by flow cytometry for DNA content. (**a**,**b**) Cell cycle histograms display the DNA content (diploid or 2N; tetraploid or 4N) after 2 cycles (48 h) of exposure and (**c**) data are expressed as the ratio of G2 to G1. Data are presented as mean ± SEM. * *p* < 0.05 for each exposure condition as it compares with the unexposed control (CTL) in the same respective time-group; ^#^ *p* < 0.05 for significant differences between O_2_-alone compared to O_2_ + IR; ^§^ *p* < 0.05 for significant differences between IR-alone compared to O_2_ + IR.

We further investigated the observed increases in DNA damage and apoptosis to determine specific alterations in cell cycle progression induced by exposure to hyperoxia and radiation. No effects on the cell cycle were observed in any of the treatment groups after 24 h of challenge exposure (Figure 4). After 48 h of hyperoxia, cells underwent G2/M arrest, as evidenced by an increase in the percentage of cells in the G2/M phase of the cell cycle with a concomitant decrease in the percentage of cells in the G1 phase of the cell cycle (Figure 4a,b). These results are quantified as the G2/G1 ratio (Figure 4c). Forty-eight hours of hyperoxia treatment resulted in a statistically significant 5.9-fold increase (*p* < 0.05) in the G2/G1 ratio. Ionizing radiation alone had no effect on the G2/G1 ratio at 48 h. However, addition of ionizing radiation to hyperoxia treatment appeared to decrease (*p* < 0.05) the degree of G2/M arrest induced by hyperoxia-alone. The combination treatment caused a significant 4.1-fold increase (*p* < 0.05) in the G2/G1 ratio, as opposed to the 5.9-fold increase induced by hyperoxia alone.

### 2.5. Modulation of Key Cell Cycle Signaling Protein Levels in Lung Epithelial Cells Exposed to Hyperoxia, Radiation, and Double-Hit Combination Challenge

**Figure 5:**
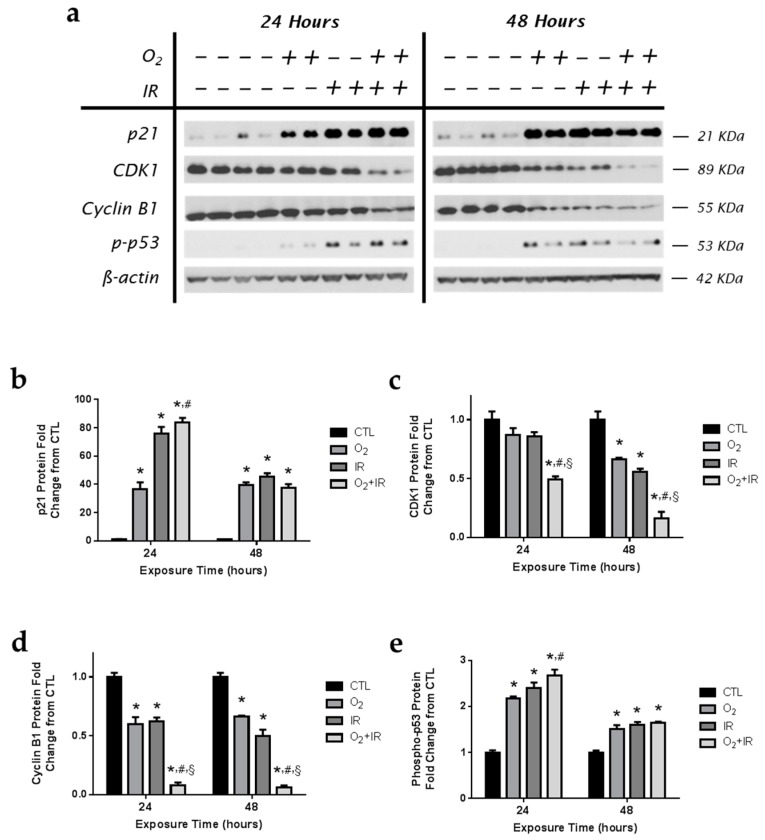
Hyperoxia and radiation exposure induce changes in cell cycle proteins. Non-tumorigenic murine alveolar type II epithelial cells (C10) were evaluated after 1 and 2 cycles of hyperoxia and radiation exposure and evaluated by (**a**) western blotting for p21, CDK1, and Cyclin B1, and phospho-p53. Densitometric analysis of band intensity for (**b**) p21; (**c**) CDK1; (**d**) Cyclin B1; and (**e**) phospho-p53 was normalized to β-actin and values are expressed as fold change from CTL. Data are presented as mean ± SEM. * *p* < 0.05 for each exposure condition as it compares with the unexposed control (CTL) in the same respective time-group; ^#^ *p* < 0.05 for significant differences between O_2_-alone compared to O_2_ + IR; ^§^ *p* < 0.05 for significant differences between IR-alone compared to O_2_ + IR.

Cell cycle signaling proteins control the cell cycle checkpoints for either transition to the next phase or cell death [18,19,20,21,22,23]. In these studies, we focused on determining the effect of hyperoxia and radiation on select key components of G2/M phase transition. Mouse pulmonary epithelial cells exposed to challenge conditions were evaluated by western blotting for p21 (Figure 5a,b); an inhibitor of the CDK1/cyclin B1 complex, CDK1 (Figure 5a,c), Cyclin B1 (Figure 5a,d), and phospho-p53 (Figure 5a,e). After just 1 cycle (24 h) of challenge exposure, increased p21 protein expression was detected for all challenge conditions and remained elevated after 48 h (Figure 5b) with increased levels of p21 among O_2_ + IR exposure (*p* < 0.05) compared to O_2_-alone after 24 h. The expression of CDK1 (Figure 5c) and cyclin B1 (Figure 5d) was significantly (*p* < 0.01) decreased in the double-hit combination challenge, compared to CTL, O_2_-alone, and IR-alone, following the first cycle of exposure and remained low after 48 h of exposure. Levels of phosphorylated p53 (Figure 5d) were elevated after 1 and 2 cycles of exposure across all challenge conditions. In the double-hit combination challenge group, the increased expression of p21 and phospho-p53 proteins, and decreased CDK1 and Cyclin B1 expression, as indices of cell cycle arrest, correlate with the observed increase in apoptotic cell death (see Figure 2b).

### 2.6. H2AX Phosphorylation due to DNA Damage in Pulmonary Epithelial Cells Exposed to Repeat Cycles of Radiation, Hyperoxia or Double-Hit Combination Challenge

**Figure 6:**
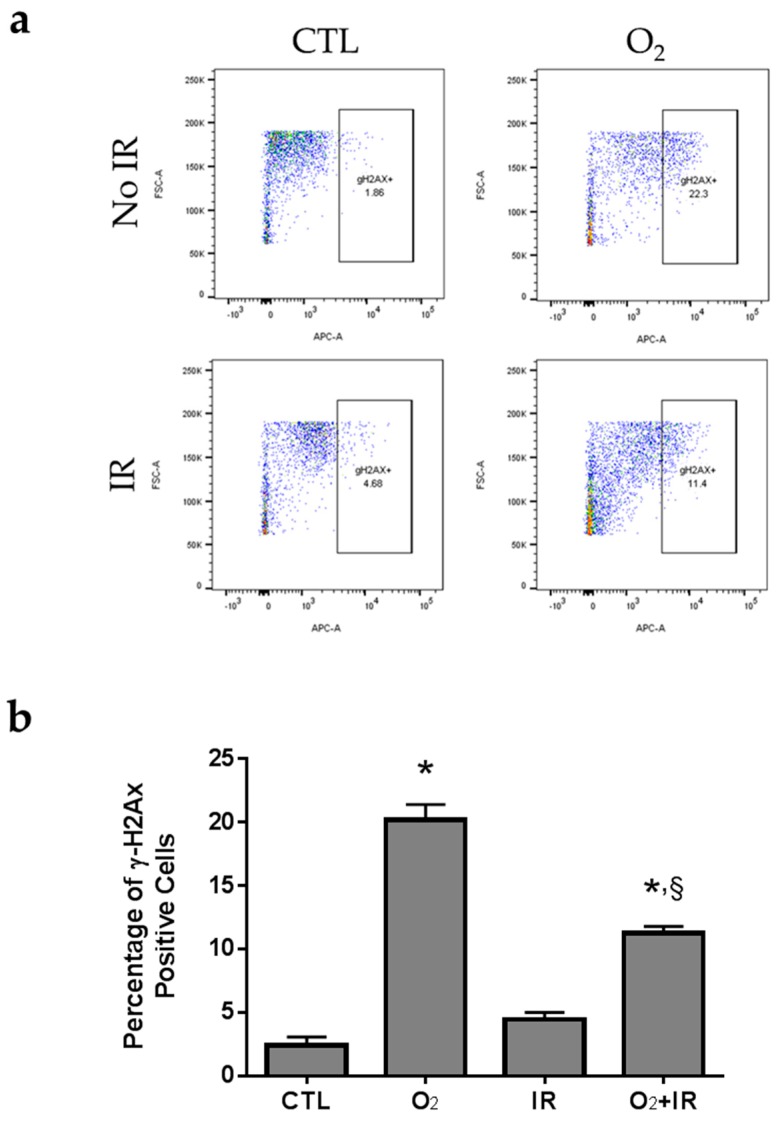
Flow cytometric analysis of H2AX phosphorylation in lung epithelial cells 48 h (2 cycles) after exposure to hyperoxia, radiation, and double-hit combination challenge. Non-tumorigenic murine alveolar type II epithelial cells (C10) were evaluated after 2 cycles of hyperoxia and radiation exposure and evaluated by flow cytometric analysis of γΗ2ΑΧ and PI. (**a**) Representative flow cytograms depicting anti-γΗ2ΑΧ (Serine 139) labeling of cells under hyperoxic conditions and IR; (**b**) Levels of H2AX phosphorylation represented by the percentages (%) of positive stained C10 cells in every cohort. Data are represented as the mean ± SEM. * *p* < 0.05 for each exposure condition as it compares with the unexposed control (CTL); ^§^ *p* < 0.05 for significant differences between IR-alone compared to O_2_ + IR.

To assess DNA double strand break damage following exposure to hyperoxia and radiation, we performed flow cytometric analysis for the detection of H2AX phosphorylation at Serine 139 (Figure 6), due to exposure of pulmonary epithelial cells to radiation, hyperoxia or the combination of the two conditions. A significant 8.4-fold increase of the H2AX phosphorylation was observed when cells were treated for 48 h or two cycles (>95% O_2_ for 8 h per cycle) of hyperoxia whereas exposure to two doses of 0.25 Gy ionizing radiation induced a 1.85-fold increase, as compared to untreated cells. Two cycles of the double-hit combination of both challenges (O_2_ + IR) led to a statistically significant increase in H2AX phosphorylation by 4.67-fold (*p* < 0.001) compared to CTL (Figure 6a,b).

### 2.7. Gene Expression Changes Related to DNA Damage and Apoptosis in Pulmonary Epithelial Cells Exposed to Repeat Cycles of Radiation, Hyperoxia or Double-Hit Combination Challenge

**Figure 7:**
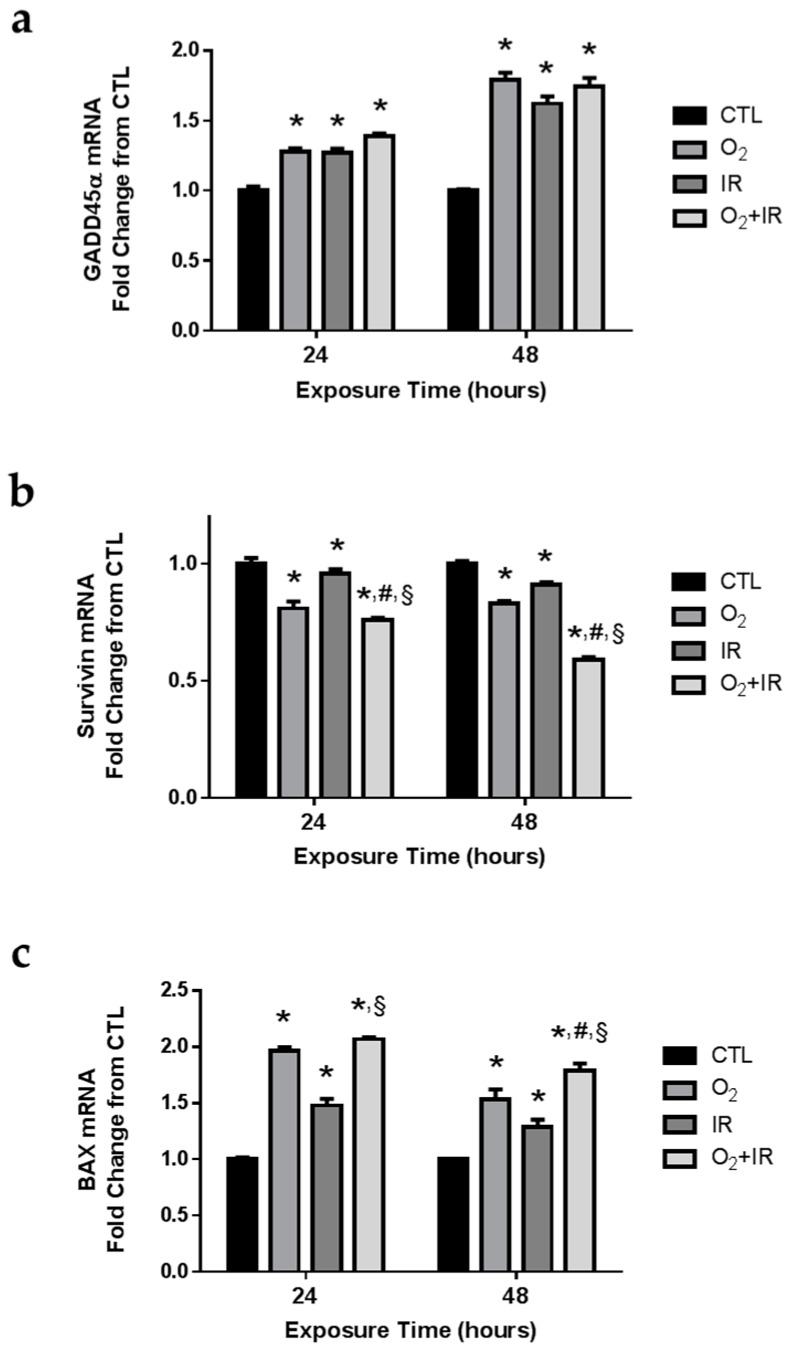
Determination of apoptosis-associated gene expression changes in lung epithelial cells exposed to hyperoxia, radiation, and double-hit combination challenge. Non-tumorigenic murine alveolar type II epithelial cells (C10) were evaluated after 1 and 2 cycles of hyperoxia and radiation exposure and evaluated by quantitative real-time PCR analysis for (**a**) *GADD45α*; (**b**) *Survivin*; and (**c**) *BAX* gene expression. Levels of target gene mRNA were normalized to 18S ribosomal RNA and values are expressed as the mean fold change from CTL. Data are presented as mean ± SEM. * *p* < 0.05 for each exposure condition as it compares with the unexposed control (CTL) in the same respective time-group; ^#^ *p* < 0.05 for significant differences between O_2_-alone compared to O_2_ + IR; ^§^ *p* < 0.05 for significant differences between IR-alone compared to O_2_ + IR.

We evaluated the expression levels of *GADD45*α (a gene encoding a protein that functions as a cellular sensor of genotoxic stress), *survivin* (a gene encoding a protein that functions as an inhibitor of apoptosis (IAP)), and *BAX* (a gene encoding a protein that functions to promote apoptosis). As an IAP, survivin is capable of binding to caspase-3 and thus negatively regulates apoptotic signaling [24]. Also, survivin gene expression is negatively regulated by p53 [25], and serves as a successful biomarker of genotoxic stress and subsequent apoptotic signaling. We asked whether gene expression changes of GADD45α, survivin, and BAX would correlate with the observation of increased DNA damage and apoptotic cell death. Indeed, all challenge conditions *in vitro* induced significant (*p* < 0.05) increases in GADD45α gene expression levels after 1 and 2 cycles of exposure (Figure 7a). Conversely, we determined significant (*p* < 0.05) decreases in survivin gene expression (Figure 7b), while increasing mRNA levels of pro-apoptotic BAX (Figure 7c). The double-hit combination challenge (O_2_ + IR) group had the highest reduction in survivin gene expression at all exposure times (24 and 48 h), with a significant (*p* < 0.05) further decrease in survivin gene expression compared to O_2_ or IR exposure alone, while the exposure to hyperoxia alone and hyperoxia plus radiation induced marked increases in BAX expression after 1 and 2 cycles of exposure.

### 2.8. Expression of Apoptotic and DNA Damage-Induced Proteins in Lung Epithelial Cells Exposed to Hyperoxia, Radiation, and Double-Hit Combination Challenge

**Figure 8:**
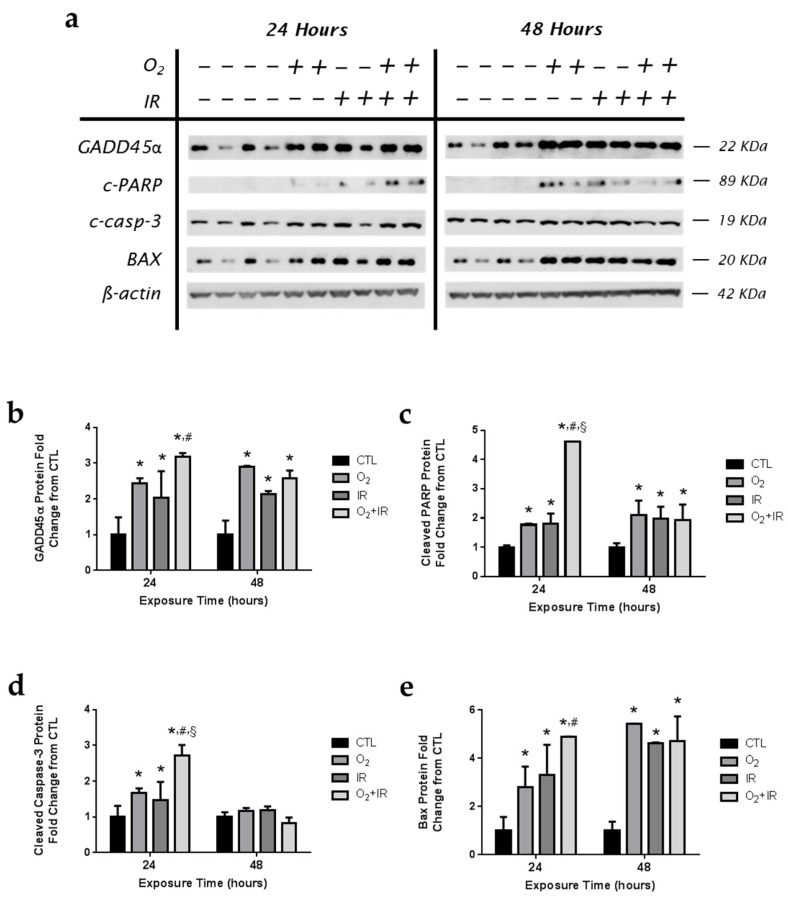
Determination of apoptosis and genotoxic stress in lung epithelial cells exposed to hyperoxia, radiation, and double-hit combination challenge. Non-tumorigenic murine alveolar type II epithelial cells (C10) were evaluated after 1 and 2 cycles of hyperoxia and radiation exposure and evaluated by (**a**) western blotting for GADD45α, cleaved-PARP, cleaved caspase-3, and BAX. Densitometric analysis of band intensity for (**b**) GADD45α; (**c**) cleaved-PARP; (**d**) cleaved caspase-3; and (**e**) BAX was normalized to β-actin and values are expressed as fold change from CTL. Data are presented as mean ± SEM. * *p* < 0.05 for each exposure condition as it compares with the unexposed control (CTL) in the same respective time-group; ^#^ *p* < 0.05 for significant differences between O_2_-alone compared to O_2_ + IR; ^§^ *p* < 0.05 for significant differences between IR-alone compared to O_2_ + IR.

Murine pulmonary epithelial cells exposed to challenge conditions were evaluated by western blotting for GADD45α (Figure 8a,b); a DNA damage-inducible protein that triggers apoptotic signaling [26,27,28], cleaved-PARP (Figure 8a,c); a key target enzyme for DNA repair following DNA damage [29,30], cleaved caspase-3 (Figure 8a,d), and BAX (Figure 8a,e). After just 1 cycle (24 h) of challenge exposure, increased GADD45α and cleaved-PARP protein expression was detected for all challenge conditions with the double-hit combination challenge of O_2_ + IR showing the greatest effect after 1 exposure cycle; significant elevations above untreated cells and O_2_ and IR exposure alone. Increased DNA damage and apoptosis were observed in the combination challenge, correlating with increased levels of cleaved-PARP and GADD45α. Levels of cleaved caspase-3, and BAX were significantly (*p* < 0.05) elevated following exposure to all challenge conditions after 1 and 2 cycles of exposure.

### 2.9. Exposure to Repeated Cycles of Hyperoxia, Radiation, and Double-Hit Combination Challenge Induces the Expression of Key Antioxidant Enzymes in Pulmonary Epithelial Cells

**Figure 9:**
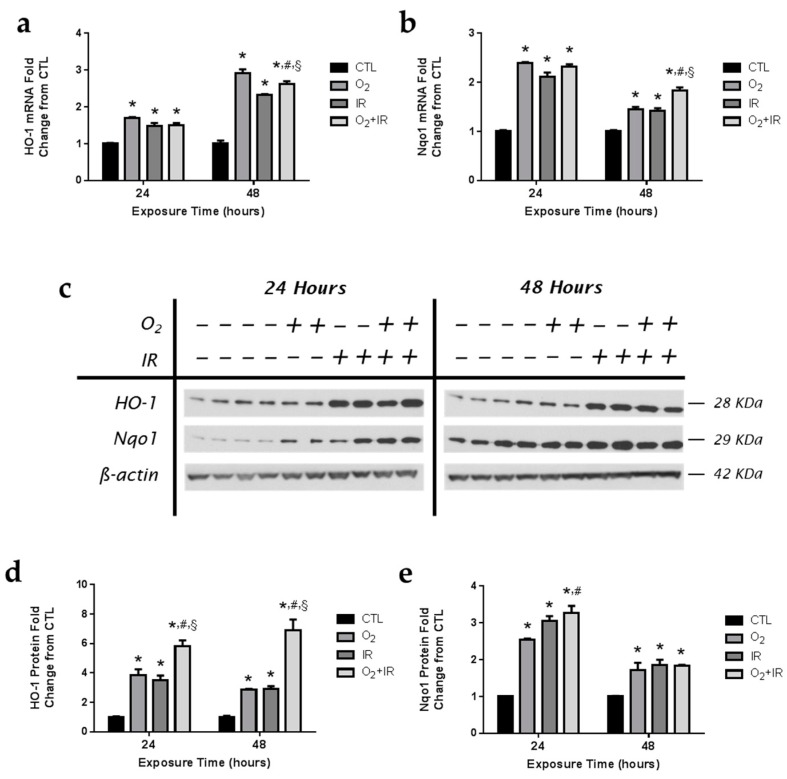
Exposure to hyperoxia, radiation, and double-hit combination challenge induces expression of antioxidant enzymes. Non-tumorigenic murine alveolar type II epithelial cells (C10) were evaluated after 1 and 2 cycles of hyperoxia and radiation exposure and evaluated by qPCR analysis of (**a**) *HO-1* and (**b**) *Nqo1* mRNA levels and by (**c**) western blotting for HO-1 and Nqo1. Levels of target gene mRNA were normalized to 18S ribosomal RNA and values are expressed as the mean fold change from CTL. Densitometric analysis of band intensity for (**d**) HO-1 and (**e**) Nqo1 was normalized to β-actin and values are expressed as fold change from CTL. Data are presented as mean ± SEM. * *p* < 0.05 for each exposure condition as it compares with the unexposed control (CTL) in the same respective time-group; ^#^ *p* < 0.05 for significant differences between O_2_-alone compared to O_2_ + IR; ^§^ *p* < 0.05 for significant differences between IR-alone compared to O_2_ + IR.

Antioxidant enzymes, such as heme oxygenase-1 (HO-1) and NADPH: quinone oxidoreductase-1 (Nqo1), are induced by select challenges that alter the oxidative state of the cell and are indicative of conditions of oxidative stress. Both hyperoxia and radiation promote the generation of free radicals and reactive oxygen species (ROS). We evaluated hyperoxia and radiation-induced changes in *HO-1* (Figure 9a) and *Nqo1* (Figure 9b) antioxidant mRNA levels in pulmonary epithelial cells after 1 and 2 cycles of exposure. Levels of *HO-1* and *Nqo1* mRNA were elevated after 1 cycle of exposure to hyperoxia and radiation. Gene expression of *HO-1* was significantly (*p* < 0.05) increased across all exposure groups, with the greatest increase in mRNA levels occurring after 48 h of exposure. Protein levels of HO-1 were significantly increased (*p* < 0.05) in all exposure groups after 1 and 2 cycles of exposure, with the greatest induction of HO-1 protein expression (5.8- and 6.9-fold increase over CTL) occurring in cells exposed to both challenge conditions (Figure 9c,d). In addition, protein levels of Nqo1 were similarly increased over control in pulmonary epithelial cells exposed to hyperoxia, radiation, and combination challenge (Figure 9c,e). Importantly, exposure to combined hyperoxia and radiation challenges led to significantly (*p* < 0.05) higher levels of both HO-1 and Nqo1 compared to single challenge exposure to hyperoxia or radiation-alone.

### 2.10. Expression of Apoptotic and DNA Damage-Induced Proteins in Lung Epithelial Cells Exposed to Hyperoxia, Radiation, and Double-Hit Combination Challenge

**Figure 10:**
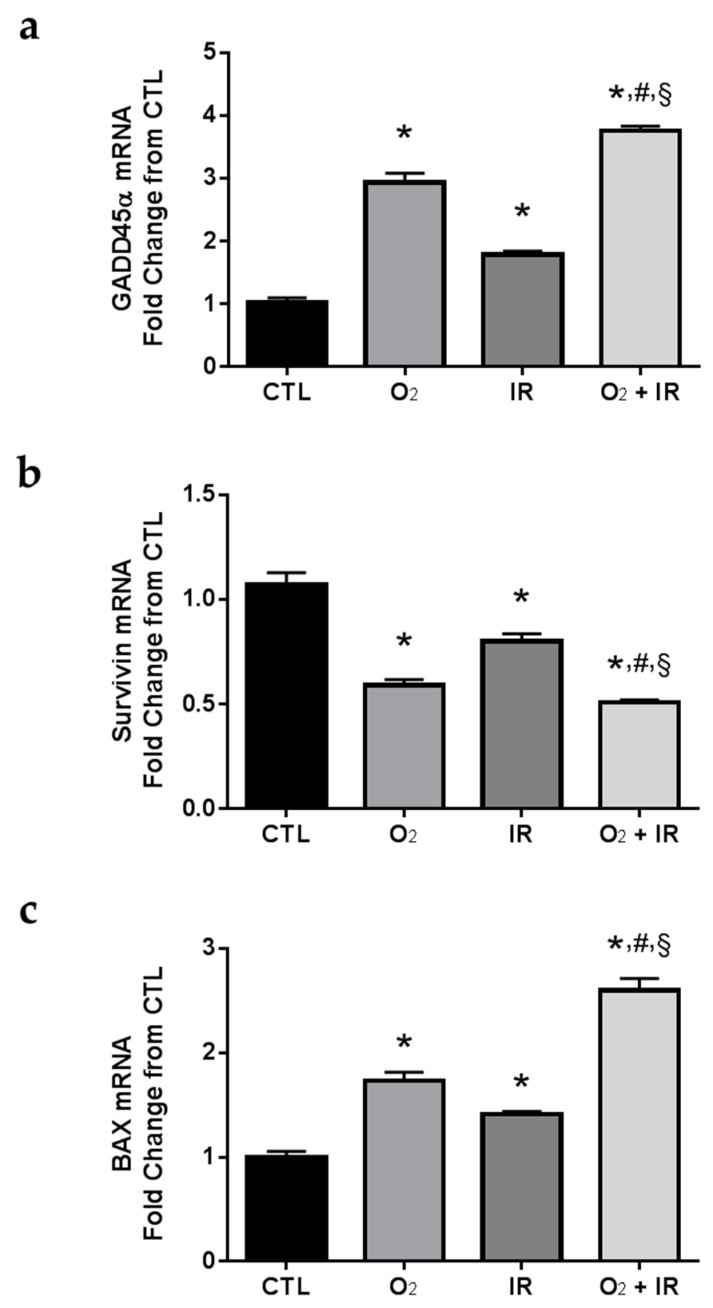
Evaluation of the effect of hyperoxia, radiation, or double-hit combination on apoptotic-relevant gene expression in murine lung tissue following 3 cycles of challenge exposure. Murine lung tissues (*n* = 3/group) were evaluated after 3 cycles of hyperoxia and radiation exposure and evaluated by quantitative real-time PCR analysis for (**a**) *GADD45α*; (**b**) *Survivin*; and (**c**) *BAX* gene expression. Levels of target gene mRNA were normalized to 18S ribosomal RNA and values are expressed as the mean fold change from CTL. Data are presented as mean ± SEM. * *p* < 0.05 for each exposure condition as it compares with the unexposed control (CTL) in the same respective time-group; ^#^ *p* < 0.05 for significant differences between O_2_-alone compared to O_2_ + IR; ^§^ *p* < 0.05 for significant differences between IR-alone compared to O_2_ + IR.

Similar to the reported findings in C10 epithelial cells exposed to similar challenge conditions, we evaluated the expression levels of *GADD45α*, *Survivin*, and *BAX* in murine lung tissues exposed to three cycles of hyperoxia (8 h) and radiation (0.25 Gy total body irradiation) exposure. Exposure to challenge conditions led to significant (*p* < 0.05) elevations in *GADD45α* and *BAX* mRNA levels (Figure 10). Importantly, the combined challenge exposure (O_2_ + IR) led to a significantly (*p* < 0.05) further increase in the expression of both *GADD45α* and *BAX* compared to exposure to hyperoxia or radiation-alone. Furthermore, mRNA levels of *Survivin* were reduced by all challenge conditions (41%, 20%, and 49% decreased from CTL exposed mice for O_2_, IR, and O_2_ + IR exposure, respectively).

### 2.11. Expression of Apoptotic and DNA Damage-Induced Proteins in Lung Epithelial Cells Exposed to Hyperoxia, Radiation, and Double-Hit Combination Challenge

**Figure 11:**
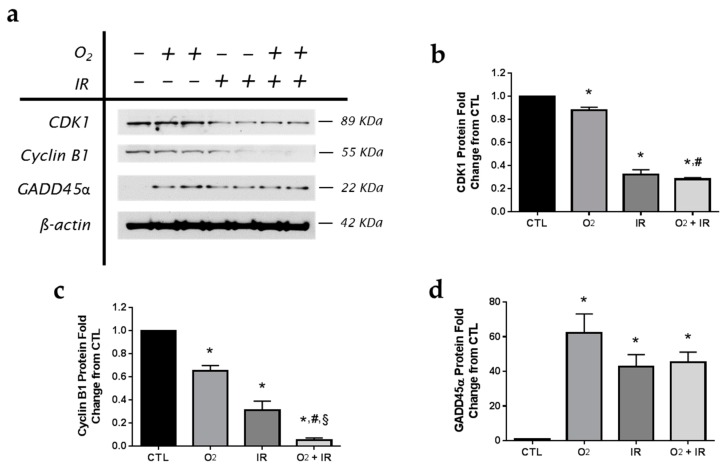
Determination of apoptotic and cell cycle proteins in murine lung tissue following exposure to repeated cycles of O_2_, IR or O_2_ + IR exposure. Murine lung tissues (*n* = 3/group) were evaluated after 3 cycles of hyperoxia and radiation exposure and evaluated by (**a**) western blotting for CDK1, Cyclin B1, and GADD45α. Densitometric analysis of band intensity for (**b**) CDK1; (**c**) Cyclin B1; and (**d**) GADD45α was normalized to β-actin and values are expressed as fold change from CTL. Data are presented as mean ± SEM. * *p* < 0.05 for each exposure condition as it compares with the unexposed control (CTL) in the same respective time-group; ^#^ *p* < 0.05 for significant differences between O_2_-alone compared to O_2_ + IR; ^§^ *p* < 0.05 for significant differences between IR-alone compared to O_2_ + IR.

We performed an *in vivo* experiment in mice to test whether our current findings in C10 lung epithelial cells correlate with the actual response of the lung to the selected stressors (O_2_, IR, and O_2_ + IR) with respect to apoptosis and cell cycle arrest. For this study, mouse cohorts (*n* = 3/group) were exposed to repeated: (a) normoxia; (b) >95% O_2_ (O_2_); (c) 0.25 Gy single fraction gamma radiation (IR); or (d) a combination of O_2_ and IR (O_2_ + IR). We decided, in order to ensure that changes are detectable in intact lungs, to expose mice to 3 cycles of the stress conditions, as opposed to the 2 cycles used in the in vitro experiments. Murine lung tissue was then evaluated for the expression of key apoptotic and cell cycle proteins (GADD45α, Survivin, Bax, CDK1, and Cyclin B1) as determined in C10 epithelial cells. Similarly to our *in vitro* results in C10 cells, murine lung (with quiescent respiratory epithelium), exposed to three cycles of hyperoxia (8 h) and radiation (0.25 Gy total body irradiation) exposure led to significant (*p* < 0.05) reductions in CDK1 (12%, 68%, and 72% decrease for O_2_, IR, and O_2_ + IR, respectively) and cyclin B1 (35%, 69%, and 95% decrease for O_2_, IR, and O_2_ + IR, respectively) cell cycle protein levels (Figure 11). Importantly, the combined challenge exposure (O_2_ + IR) led to significantly (*p* < 0.05) further reductions in both proteins compared to exposure to hyperoxia-alone. Furthermore, levels of GADD45α were equally elevated across all challenge conditions (62-, 43-, and 45-fold increased over CTL exposed mice for O_2_, IR, and O_2_ + IR exposure, respectively).

## 3. Discussion

We have recently identified possible pulmonary toxicity associated with space travel by the use of a cell system to model repeated individual or combined stressors such as radiation or high oxygen levels associated with space exploration [12]. Our findings from the current *in vitro* model system of similar cycling levels of hyperoxia/radiation exposure identified damage at the lung cellular level, showing marked increases in DNA damage and apoptosis, and decreases in cell survival that were especially profound in the double-hit combination challenge group. Cells exposed to O_2_ and IR challenges also displayed increased phosphorylation of H2AX along with elevated levels of DNA damage-induced proteins, cleaved-PARP and GADD45α proteins, correlating with decreased survivin and increased *GADD45*α and *BAX* gene expression. The significant levels of epithelial cell death following exposure to radiation and hyperoxia detected in this study attributed to increased apoptosis, cell cycle arrest, and increased oxidative stress provides, in part, an explanation for the tissue damage and toxicity observed in the lungs of animals exposed to similar cycling exposures to the same challenges [12,13]. The information presented here, in combination with our *in vivo* findings, provides useful information applicable to ongoing research relevant to human space missions.

Our *in vitro* model of double-hit exposure revealed that double-hit combination challenge results in increased cell death and apoptosis as compared to either hyperoxia or IR alone. Pulmonary epithelial cells exposed to the double-hit challenge also expressed increased levels of cleaved-PARP, GADD45α cleaved caspase-3, and BAX. The increase in cleaved-PARP and GADD45α proteins was observed after exposure to 1 and 2 cycles (24 and 48 h) of challenge conditions in pulmonary epithelial cells, especially among cells exposed to combination treatment. Increased GADD45α expression also correlated with increased DNA damage and apoptotic cell death indicating GADD45α induced activation of caspase-3 through cellular increases in BAX. At the same time, the level of cell cycle arrest protein p21 was elevated, while the levels of CDK1 and cyclin B1 were decreased in cells exposed to O_2_ and IR. Accumulation of p21 inhibits G2/M transition leading to cell cycle arrest, which was observed after 2 cycles of exposure among cells exposed to hyperoxia and hyperoxia plus radiation [18]. Activation of CDK1-Cyclin B1 complex is necessary for cell cycle G2/M transition. We acknowledge a discrepancy between the findings from the western blot analysis of cell cycle proteins, such as p21 and phospho-p53, and the cell cycle arrest analysis determined by flow cytometry. Specifically, flow cytometry showed that exposure to IR after 24 and 48 h did not alter cell cycle; however, western blot evaluation showed that upon IR exposure, both p21 and phosphor-p53 were both induced. According to our data, although the dose of radiation exposure (0.25 Gy for 1 cycle and 0.50 Gy for 2 cycles of exposure) is able to induce an elevation in phospho-p53 and p21, it is not able to induce cell-cycle arrest as determined by DNA content using flow cytometry. The extent of cellular damage following exposure to such low radiation levels (0.25 or 0.50 Gy) may not be sufficient to induce cell cycle arrest. However, such exposure may lead to the phosphorylation of cell cycle proteins, which we have indeed identified by western blot analysis.

It is known that p53 holds a regulatory role not only for the G1 phase of the cell cycle, but also for the G2/M phase, which has been reviewed by Taylor and Stark [31]. Thus phospho-p53 and subsequently p21, a downstream activated protein can be used as markers of cell cycle arrest in G2/M phase of the cell cycle as well. These studies warrant additional investigation to further evaluate lung epithelial cell cycle arrest and cellular apoptosis relevant to the double-hit condition and to identify potential countermeasures of this damage as described in a recent review by Schmidt and Goodwin [6]. Indeed, pathological changes in hyperoxic lungs, for example, are associated with lung cell death via apoptosis or necrosis [32,33,34,35]. Similarly, IR can directly target critical cellular macromolecules such as DNA, proteins and lipids. DNA damage by radiation that cannot be repaired condemns the cell to reproductive death by either apoptosis, mitotic catastrophe or senescence [36].

**Figure 12:**
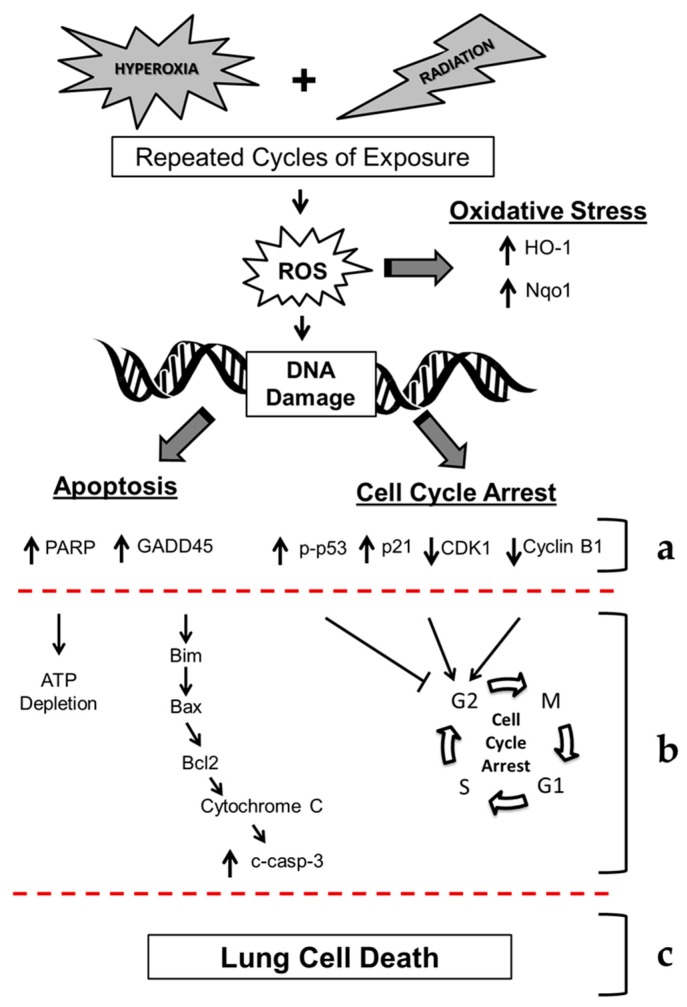
Mechanism of cell death in murine lung epithelial cells after repeated cycles of hyperoxia, radiation, and double-hit combination challenge. Hyperoxia and radiation exposure generates reactive oxygen species (ROS) that lead to increases in DNA damage. Oxidative stress induces the expression of cytoprotective enzymes (HO-1 and Nqo1). We observed significant increases in molecular markers of DNA damage (γ-H2AX, GADD45α and cleaved-PARP), along with a key upstream executioners of apoptosis (cleaved caspase-3). (**a**) Additionally, we observed increased Annexin-V, a marker of apoptosis, and levels of apoptotic proteins (cleaved PARP, GADD45α, and cleaved caspase-3) and altered levels of cell signaling proteins (phopso-p53, p21, CDK1 and cyclin B1). Therefore, we propose that hyperoxia and radiation exposure (**b**) increase apoptosis (via ATP depletion and activation of the intrinsic apoptotic pathway) and G2/M cell cycle arrest, ultimately leading to (**c**) lung cell death. **↑** indicates an increase in gene expression or protein levels; ↓ indicates a decrease in gene expression or protein levels.

Based on the data presented in this *in vitro* study, as well as in our previous *in vivo* study of double-hit combined challenge of hyperoxia and radiation, we present the proposed mechanism of hyperoxia plus γ-irradiation-induced lung cell death (see Figure 12). It is known that hyperoxia and γ-irradiation produce ROS and reactive nitrogen species (RNS) and results in DNA damage [12]. Our current findings show increased phosphorylation of γ-H2AX, indicative of increased DNA damage. The double-hit challenge induces both the apoptotic response as well as the cell cycle arrest response, while exposure to hyperoxia or radiation alone increased the number of necrotic cells. Our results show an increased level of cleaved-PARP in challenged cells. Increased activation of PARP would lead to increased utilization of ATP for poly(ADP-ribosyl)ation reactions to facilitate DNA replication, however, resulting in depletion of cellular ATP that further leads to cell death. We have also observed apoptosis and cell death in our *in vitro* model confirming that PARP activation may contribute to cell death following exposure to hyperoxia and radiation. Simultaneously, increased expression of GADD45α (a DNA-damage inducible protein) [26,27,28], as observed in this current study, would lead to dissociation of Bim (an apoptotic protein) from microtubule associated components and translocate to mitochondria. Once in the mitochondria, Bim interacts with Bcl-2 and results in relieving BAX that leads to the release of cytochrome c from the mitochondria to the cytosolic compartment where it activates procaspase-9 to active caspase-9, the initiator of programmed cell death. Activated caspase-9 then activates procaspase-3 to caspase-3 that acts on the CAD/ICAD complex (Caspase-activated DNase/inhibitor of CAD) to release CAD, which translocates to the nucleus and results in the breaking down of nuclear DNA at specific sites and subsequent cell death. Therefore, increased levels of GADD45α, BAX, and cleaved caspase-3, as seen in our current studies, may signify a novel mechanism by which double-hit radiation/hyperoxia exposure of cells leads to DNA damage and ultimately cell death.

In our current study under the challenge condition, the expression of p21 protein increased while the expression CDK1 and Cyclin B1 proteins decreased. The increase in the cell cycle signaling protein p21 following exposure to hyperoxia and radiation led to cell cycle transition arrest at G2/M. It is well known that p21 is an inhibitor of the CDK1-Cyclin B1 complex [18]. We have observed increased expression of p21 and decreased expression of CDK1 and Cyclin B1 following exposure to hyperoxia and radiation. Therefore, such an increase in p21 and a simultaneous decrease in CDK1 and cyclin B would result in cell cycle arrest and cell death. In our current study, we have observed increased apoptosis, DNA damage, and oxidative stress following exposure to hyperoxia and radiation challenge conditions, which may be a potential mechanism of EVA-relevant cell damage leading to cell death (see Figure 12). We acknowledge that several other factors may contribute to cell death observed in the double-hit combination challenge as described extensively by Cucinotta and co-workers [37,38,39]. Specifically it would be important to investigate DNA repair mechanisms and how they relate to the observed cell damage and death seen in our *in vitro* model system.

Additionally, we acknowledge the use of proliferating C10 murine pulmonary type II epithelial cells, which were undergoing proliferation during experimentation, despite the quiescent state of the respiratory epithelium as a potential limitation of the current study findings. Furthermore, while the current findings represent only a cell model of investigating the consequences of radiation and hyperoxia to the whole lung, the importance of specifically investigating the lung epithelium and molecular mechanisms underlying such exposures is warranted. Exposure to sublethal levels of high oxygen causes damage to all cell structures of the alveolus including damage to nearly 50% of the lung capillary endothelial cells [15]. For this reason, in our recent work by Velalopoulou *et al*. [40] we evaluated radiation exposures in other relevant lung cell types. However, exposure to hyperoxia are initially detected by the epithelial lining of the airways, which prompted us to use this cell type to evaluate relevant pathways that might be involved. It is, however, known by the work of many investigators, that endothelial and interstitial cells are also affected by high oxygen levels [41]. In fact, work in rats and baboons exposed to high oxygen (40%–60%), showed an increase in interstitial cells, quantitative evidence of injury to alveolar epithelial cells, and a significant fall in the number of capillary endothelial cells during the late phases of hyperoxic lung injury.

The current Design Reference Missions (DRMs) for planetary exploration detailed by NASA include repeated cyclic exposures to hypobaric mild hypoxia at 8.2 psia/34% O_2_ (~4500 ft. elevation) for 18 h mixed with exposure to hypobaric hyperoxia at 4.3 psia/100% O_2_ for ~6 h (suit) [6]. This regime will be repeated daily over a two to three week period during exploration DRMs and these conditions have not been characterized in any animal model or in humans. While the current model system outlined above is robust, utilitarian and capable, it lacks two essential requirements to adequately test the DRMs defined by the National Aeronautics and Space Administration (NASA). First the ability to simulate microgravity and second the longevity to be maintained over weeks and even months. Thus, in the future we plan to pursue experimentation in a 3 dimensional (3D) model of human lung developed by our colleagues at the NASA Johnson Space Center. This complex model of human lung propagated in 3D is well characterized [42,43] and presents with the typical biomarkers expected of normal human lung during prolonged culture (>3 months). Additionally the model is developed in a modeled microgravity environment reflective of conditions seen in actual space habitation. This system is easily manipulated to test potential countermeasures to the combined effects of cyclic hypobaric O_2_ concentrations (both hypoxic and hyperoxic), microgravity, and various IR exposures. Further, as the system embodies considerable longevity, repeated IR doses may be employed to observe damage and implement protective strategies by dose-dependent countermeasure application.

## 4. Materials and Methods

### 4.1. Experimental Plan of in Vitro Cell Exposure

Non-tumorigenic murine alveolar type II epithelial cells (C10), a kind gift of Alvin Malkinson, University of Colorado School of Pharmacy [44] were used as surrogates for alveolar epithelial cell injury, as we have previously reported [40]. C10 murine alveolar epithelial Type II cells were grown in 4 mL of Dulbecco’s modified Eagle’s medium (4500 mg/L glucose; Invitrogen, Carlsbad, CA, USA) containing 10% fetal bovine serum (Atlanta Biologicals, Norcross, GA, USA), penicillin (100 units/mL), streptomycin (100 mg/mL), and l-Glutamine (2 mM) (Life Technologies, Inc., Gaithersburg, MD, USA). Cells were exposed to: (a) normoxia (CTL); (b) >95% O_2_ (O_2_); (c) 0.25 Gy ionizing γ-radiation (IR); or (d) a combination of >95% O_2_ and 0.25 Gy ionizing γ-radiation (O_2_ + IR). These conditions were similar to our *in vivo* murine model of double-hit exposure to hyperoxia and radiation [12]. Cells were exposed to hyperoxic conditions for 8 h followed by normoxia (ambient air containing 5% CO_2_) for 16 h representing 1 cycle of exposure. Cells were harvested after 1 cycle (24 h) and 2 cycles (48 h) of exposure.

### 4.2. Exposure of Lung Epithelial Cells to Radiation

Non-tumorigenic murine alveolar type II epithelial cells (C10) were irradiated with a Shepherd Mark 1 ^137^Cs irradiator delivering a dose of 1.0 Gy/minute. Cells were plated in coated 6 cm Petri dishes (Nalgene-Nunc, Thermo-Fisher, Rochester, NY, USA) to 80% confluency, placed in aluminum chambers, and sealed under a controlled atmosphere (described below). These chambers allow full exposure of cells to gamma rays. The scheme displayed in Figure 1a shows details of the *in vitro* procedure.

### 4.3. Exposure of Lung Epithelial Cells to Hyperoxia

Non-tumorigenic murine alveolar type II epithelial cells (C10) [45] were plated in sterile, vented Permanox™ solvent-resistant plastic 60 mm × 15 mm cell culture dishes (Permanox, Nunc International, Rochester, NY, USA) and enclosed in specially-designed O-ring sealed, leak-proof aluminum chambers (see Figure 1b) [46]. The atmosphere inside the chambers can be changed to any desired oxygen content through a system of valves and an exposure manifold, as described [46]. Chambers were kept at 37 °C at all times. Duration of exposure to hyperoxic conditions was for 8 h followed by normoxia (ambient air containing 21% O_2_ and 5% CO_2_) for 16 h representing 1 cycle of exposure. Cells were harvested after 1 cycle (24 h) and 2 cycles (48 h) of exposure. Work by Allen *et al*. [47], noted that while it takes just minutes for the air inside a petri dish to equilibrate with the air above it, it takes a few h for the oxygen content in the medium in the tissue culture flasks and around it to achieve equilibrium. We incubated our cells under hyperoxic conditions for 8 h, which ensured adequate oxygen solubility in the medium. We did not detect any color changes in the medium with oxygen exposure, a sign of pH change. We therefore, assumed that no modification of the medium was in effect.

### 4.4. Western Blotting

Immunoblot analysis on lung epithelial cell lysates was performed as previously described [48] using primary antibodies against cleaved-poly (ADP-ribose) polymerase (PARP), growth arrest and DNA damage protein (GADD45α), phospho-53, p21, cyclin-dependent kinase 1 (CDK1), Cyclin B1, cleaved caspase-3, Bcl-2-associated X protein (BAX), heme oxygenase-1 (HO-1), and NADPH: quinone oxidoreductase-1 (Nqo1) (Cell Signaling Technology, Danvers, MA, USA). Densitometric analysis of western blots with β-actin normalization of protein expression was performed using Gel-Pro Analyzer software (Version 6.0, MediaCybernetics, Silver Spring, MD, USA). Briefly, cells were lysed in phosphate buffered saline (PBS) containing protease inhibitors. Immunoblot analysis of cell lysates was then performed using 12 well SDS 12% NuPAGE gel (Invitrogen, Carlsbad, CA, USA). Electrophoresis was performed at 200 volts for 1 h. Transfer to PolyScreen PVDF transfer membrane (PerkinElmer Life Sciences, Boston, MA, USA) was performed for 1 h at 25 volts at room temperature. Membrane was blocked overnight in 5% non-fat dry milk in PBS. The non-fat dry milk was then discarded and the membrane was incubated with primary antibody. Protein levels of cleaved-PARP, GADD45α, phospho-p53, p21, CDK1, cyclin B1, cleaved caspase-3, BAX, HO-1, and Nqo1 were detected using manufacturer recommended dilutions (Cell Signaling Technology, Danvers, MA, USA). The membrane was washed five times and then incubated in secondary antibody conjugated to horseradish peroxidase for 45 min at room temperature. Membranes were developed using Western Lighting Chemiluminescence Reagent Plus (PerkinElmer Life Sciences, Boston, MA, USA) and quantified by densitometric analysis of specific bands (89 kDa for cleaved-PARP, 22 kDa for GADD45α, 53 kDa for phospho-p53, 21 kDa for p21, 34 kDa for CDK1, 55 kDa for cyclin B1, 17/19 kDa for cleaved caspase-3, 20 kDa for BAX, 28 kDa for HO-1, and 29 kDa for Nqo1) that were adjusted for loading using β-actin expression levels.

### 4.5. Lung Epithelial Cell Counts

Cell counting was performed using an inverted Olympus IX-51-microscope fitted with a BASLER scA1300-32fm monochrome digital camera. This setup was connected to a PC equipped with Image J software (National Institutes of Health, Bethesda, MD, USA; Available online: http://imagej.nih.gov/ij/; 1997–2016). Treated cells growing in 6 cm Petri dishes were stained with Crystal Violet (Sigma, St. Louis, MO, USA). Dishes were mounted onto the microscope stage equipped with a special holder and photographed. Images were captured from 5 randomly-selected fields per dish at 200× magnification. Captured images were processed using the cell counting function of Image J.

### 4.6. Comet Analysis

Non-tumorigenic murine alveolar type II epithelial cells (C10) were challenged with hyperoxia and/or radiation and processed for comet assay analysis as per manufacturer’s instructions (Trevigen, Gaithersburg, MD, USA). Briefly, cells (1 × 10^5^ cells/mL in PBS) were mixed with LMAgarose^®^ (1:10, *v*/*v*) and immediately pipetted onto Trevigen’s CometSlides™. Cells were then lysed (4 °C, 30 min) and kept in dark for unwinding at room temperature. Electrophoresis was done in a horizontal electrophoresis unit at 18 volts (300 mA) for 25 min. Slides were washed twice with deionized water, fixed in 70% ethanol and dried at 45 °C. DNA was stained by SYBR green (Trevigen). At least 150 cells were scored per group. Visual analysis of cells and comet tail length was measured using Comet Image Analysis software (Comet Assay IV, Perceptive Instruments Ltd., Haverhill, UK). Images were captured on an Olympus IX51 fluorescence microscope using a monochrome CCD FireWire camera (Perceptive Instruments Ltd., Haverhill, UK).

### 4.7. Gene Expression Analysis

Quantitative Polymerase Chain Reaction (qPCR) was performed using TaqMan^®^ Probe-Based Gene Expression Assays supplied by Applied Biosystems, Life Technologies (Carlsbad, CA, USA). Individual TaqMan gene expression assays were selected for *GADD45α*, *Survivin* (*Birc5: baculoviral inhibitor of apoptosis repeat-containing 5*), and *Bcl-2-associated X protein* (*BAX*). Briefly, cells were lysed and RNA was isolated using a commercially available kit, QIAprep Spin Miniprep Kit, supplied by Qiagen (Valencia, CA, USA). Total RNA was quantified using a NanoDrop 2000 (ThermoFisher Scientific, Waltham, MA, USA). Reverse transcription of RNA to cDNA was then performed on a Veriti^®^ Thermal Cycler using the High Capacity RNA to cDNA kit supplied by Applied Biosystems, Life Technologies (Carlsbad, CA, USA). Quantitative real-time PCR was performed using 50 ng of cDNA per reaction well on a StepOnePlus™ Real-Time PCR System (Applied Biosystems, Life Technologies, Carlsbad, CA, USA). Gene expression data was normalized to 18S ribosomal RNA housekeeping gene and calibrated to the control samples according to the 2^−ΔΔ*C*t^ method as previously described [40,49,50].

### 4.8. Analysis of Cell Cycle by Flow Cytometry

Non-tumorigenic murine alveolar type II epithelial cells (C10) exposed to CTL, O_2_, IR, or O_2_ + IR were trypsinized, washed with 1% FBS in PBS, resuspended in PBS, and fixed in ice-cold ethanol. After fixation, cells were washed with 1% FBS in PBS, resuspended in PBS, treated with phosphate citric acid buffer (192 mM Na2HPO4; 4 mM citric acid, pH 7.8), and stained in PI/RNase buffer (50 μg/mL propidium iodide, 267 μg/mL RNase A, 1% FBS in PBS). A FACSCalibur flow cytometer (BD Biosciences, San Jose, CA, USA) was used to measure the DNA content of 20,000 cells per sample. Cell cycle analysis was performed with FlowJo V10.1 software (Ashland, OR, USA).

### 4.9. Analysis of Apoptosis/Necrosis by Flow Cytometry

For the evaluation of apoptotic and necrotic death by flow cytometry, cells were exposed to 2 cycles (48 h) of hyperoxia, and/or radiation and harvested following trypsinization and centrifugation, at 1500 rpm, for 5 min, at 4 °C. Cells were stained by using Abcam’s Annexin V-FITC Apoptosis Detection Kit (ab14085, Abcam, Cambridge, MA, USA) according to the manufacturer’s instructions. Briefly, cells were incubated with 500 μL of 1× Binding Buffer, 5 μL Annexin V-FITC and 5 μL propidium iodide (PI) for 10 min in the dark, at room temperature. Samples were analyzed in a FACSCanto system (BD Biosciences, San Jose, CA, USA) and the data were processed using FlowJo V10.1 software.

### 4.10. Analysis of H2AX Phosphorylation by Flow Cytometry

For the cytometric analysis of H2AX phosphorylation (on serine 139), cells exposed to 2 cycles (48 h) of hyperoxia, and/or radiation and harvested in DMEM, and washed in cold PBS twice, at 1500 rpm, at 4 °C, for 5 min. Cell pellet was resuspended in ice cold solution of 70% ethanol and the samples were incubated at −20 °C for 1 h. Cells were washed twice in cold FACS buffer (10% FBS, 0.1% NaN3 in PBS) and collected by centrifugation. H2AX phosphorylation was evaluated by incubating C10 cells with Alexa Fluor^®^ 647 anti-H2A.X-Phosphorylated (Ser139) Antibody (Biolegend, San Diego, CA, USA), overnight at 4 °C following the manufacturer’s instructions for the appropriate dilution, in FACS buffer. Next day, cells were washed three times in cold FACS buffer and the cells were resuspended at 0.5 × 10^7^–1 × 10^7^ cells/mL of FACS buffer. Samples were kept on ice and protected from light until analysis in a FACSCanto system (BD Biosciences, San Jose, CA, USA). Data were analyzed using FlowJo V10.1 software.

### 4.11. In Vivo Animal Exposure Study Design

Our studies used female C57/BL6 mice exposed to radiation, hyperoxia, or combination challenge, as previously described [12,13]. Mice were obtained from Charles River (Wilmington, MA, USA) and irradiated at 6–8 weeks of age under animal protocols approved by the Institutional Animal Care and Use Committee (IACUC) of the University of Pennsylvania. Animals were housed in conventional cages under standardized conditions with controlled temperature and humidity and a 12–12 h day-night light cycle. Animals had free access to water and chow (Semipurified AIN-93G diet, Test Diet, Bloomsburg, IN, USA). For this study mouse cohorts (*n* = 3/group) were exposed to: (a) normoxia; (b) >95% O_2_ (O_2_); (c) 0.25 Gy ionizing gamma radiation (IR); or (d) a combination of >95% O_2_ and 0.25 Gy ionizing gamma radiation (O_2_ + IR). Mice were exposed to 3 cycles of challenge conditions and euthanized for lung tissue evaluation.

Mouse irradiation was performed with a Gammacell 40 137Cs irradiator (Atomic Energy of Canada Limited, Ottawa, ON, Canada) as described in our previous work [12,13]. During irradiation, the animals were held in a circular, well-ventilated custom-made Plexiglas container that minimized their movement so that the whole body would uniformly receive the radiation dose. Mice exposed to 0 Gy served as sham controls. The average dose rate was 0.43 Gy per minute and was corrected for decay each day. The delivered dose was 0.25 Gy given 3 times a week (Monday, Wednesday, and Friday). Mice were immediately placed in hyperoxic conditions for 8 h following irradiation. Mice were exposed to a continuous flow of pO_2_ at 10 L/min in micro-isolator cages after removing the lids and placing the cages in a sealed Plexiglas chamber that allowed the simultaneous exposure of 6 mouse cages, yielding O_2_ concentrations of 95%–100% for 8 h followed by intervening normoxia (ambient air containing 21% O_2_ and 5% CO_2_).

### 4.12. Statistical Analysis

All data were analyzed using two-way analysis of variance (ANOVA) to test for the main effects of time and challenge (O_2_, IR, and O_2_ + IR) exposure on measured parameters. Post-tests (Tukey’s multiple comparisons tests) were conducted analyzing significant differences among treatment groups (CTL, O_2_, IR, and O_2_ + IR) within each harvest time point (24- and 48 h). Statistically significant differences were determined using GraphPad Prism version 6.00 for Windows, GraphPad Software (La Jolla, CA, USA; Available online: www.graphpad.com). Results are reported as mean ± the standard error of the mean (SEM). Levels of target gene mRNA are reported as the mean fold change from CTL at each of the respective time points (24 and 48 h) ± SEM. Statistically significant differences were determined at *p*-value of 0.05. Asterisks shown in figures indicate significant differences between exposure groups (O_2_, IR, and O_2_ + IR) and unexposed non-tumorigenic murine alveolar type II epithelial cells (CTL). ^#^ shown in figures indicate significant differences between O_2_-alone compared to O_2_ + IR. ^§^ shown in figures indicate significant differences between IR-alone compared to O_2_ + IR.

## 5. Conclusions

The current study aimed to provide novel evidence relevant to cell damage associated with lung complications from space exploration by developing a novel cell model system of combined stressors such as radiation and high oxygen levels. We have characterized a novel *in vitro* model system to study the effects of repeated double-hit exposure to low-level radiation and hyperoxia in lung epithelial cells. We have identified mechanisms of cell damage leading to cell death such as activation of apoptotic pathways, induction of cell cycle arrest, and a notable increase in oxidative stress that would be useful for identifying risks and testing potential countermeasure strategies for mitigating lung injury associated with prolonged space travel.

## Abbreviations

BAX	Bcl-2-associated X protein
Casp-3	caspase-3
Casp-9	caspase-9
CDK1	Cyclin-dependent kinase 1
CTL	control
DCI	decompression Illness
EVA	extravehicular activity
GADD45α	growth arrest and DNA damage
HO-1	heme oxygenase-1
IAP	inhibitor of apoptosis
Nqo1	NADPH: quinone oxidoreductase-1
PARP	poly(ADP-ribose) polymerase
PBS	phosphate buffered saline
qPCR	quantitative polymerase chain reaction
RNS	reactive nitrogen species
ROS	reactive oxygen species
SEM	standard error of mean
